# Commissioning of Aktina SRS cones and dosimetric validation of the RayStation photon Monte Carlo dose calculation algorithm

**DOI:** 10.1007/s13246-023-01315-7

**Published:** 2023-08-21

**Authors:** Andy Schofield, Matthew Newall, Dean Inwood, Simon Downes, Stéphanie Corde

**Affiliations:** 1 Radiation Oncology Department, Prince of Wales Hospital, Randwick, NSW 2031 Australia; 2 Centre for Medical Radiation Physics, University of Wollongong, Wollongong, NSW 2522 Australia; 3 Illawara Health and Medical Research Institute, University of Wollongong, Wollongong, NSW 2522 Australia

**Keywords:** Radiotherapy, Stereotactic cones, Aktina, Monte Carlo, RayStation, Beam modelling

## Abstract

Clinical implementation of SRS cones demands particular experimental care and dosimetric considerations in order to deliver precise and safe radiotherapy to patients. The purpose of this work was to present the commissioning data of recent Aktina cones combined with a 6MV flattened beam produced by an Elekta VersaHD linear accelerator. Additionally, the modelling process, and an assessment of dosimetric accuracy of the RayStation Monte Carlo dose calculation algorithm for cone based SRS was performed. There are currently no studies presenting beam data for this equipment and none that outlines the modelling parameters and validation of dose calculation using RayStation’s photon Monte Carlo dose engine with cones. Beam data was measured using an SFD and a microDiamond and benchmarked against EBT3 film for cones of diameter 5–39 mm. Modelling was completed and validated within homogeneous and heterogeneous phantoms. End-to-end image-guided validation was performed using a StereoPHAN™ housing, an SRS MapCHECK and EBT3 film, and calculation time was investigated as a function of statistical uncertainty and field diameter. The TPS calculations agreed with measured data within their estimated uncertainties and clinical treatment plans could be calculated in under a minute. The data presented serves as a reference for others commissioning Aktina stereotactic cones and the modelling parameters serve similarly, while providing a starting point for those commissioning the same TPS algorithm for use with cones. It has been shown in this work that RayStation’s Monte Carlo photon dose algorithm performs satisfactorily in the presence of SRS cones.

## Introduction

Linear accelerator (linac) based stereotactic radiosurgery (SRS) treatments are achievable on non-dedicated stereotactic linacs via the attachment of high definition multileaf collimators (MLC) or cone collimators to the treatment head [1]. Of particular advantage over MLC based treatments, in this setting, cones provide a sharper penumbra, lower transmission, better mechanical stability due to fewer moving parts, and a beam model optimised for each cone [1].

The acquisition of beam data needed for the creation of a beam model with cones demands high geometric accuracy and special dosimetric considerations. Before the release of IAEA’s code of practice for small field dosimetry [2], there were inconsistencies in set-up conditions for the measurement of small field output factors and particular concerns with regards to the detector-specific corrections needed for small fields [2–4].

Dose calculation accuracy for small fields in radiotherapy demands proper modelling of secondary electrons and therefore model-based algorithms are preferred. Within this category of algorithms, Monte Carlo and deterministic methods (grid-based Boltzmann solvers) are considered the most accurate dose calculation methods available [3]. The main issue is in the context of lateral electron scattering in heterogeneous media and conditions of lateral electron disequilibrium (LED) [1], [3].

A Monte Carlo photon algorithm was introduced within the version 8B software release of the RayStation (RaySearch Laboratories AB, Stockholm, Sweden) treatment planning system (TPS) that is calculated directly on the GPU and based on the EGSnrc code system [5]. Richmond et al. validated the accuracy of RayStation’s photon Monte Carlo implementation (version 10 A) in heterogeneous media for a Varian TrueBeam linac and open MLC fields [6]. Other authors have validated RayStation’s Collapsed Cone Convolution algorithm for open fields [7, 8]and Yongsook et al. included Varian stereotactic cones as part of their commissioning for a flattening filter free (FFF) beam (versions 3, 5, and 8 A respectively) [9]. To this end, there currently appears to be no literature concerning beam data collection, modelling, and validation of the RayStation Monte Carlo dose calculation algorithm for SRS treatment using Aktina stereotactic cones (Congers, NY, USA) and an Elekta VersaHD linac (Stockholm, Sweden).

This article aims to present beam commissioning data for a 6 MV flattened beam produced by an Elekta VersaHD linac and Aktina cones of sizes ranging from 5 to 39 mm in diameter. The modelling process using RayStation’s version 9B Monte Carlo dose engine is described before validation results are presented for homogeneous and heterogeneous phantoms. Finally, an investigation of typical calculation time is made.

## Methods

### Beam data collection

The RayStation treatment planning system (TPS) requires a specific set of data to create a new beam model. Specifically, for stereotactic cones, this includes percentage depth dose (PDD) scans, dose profiles at multiple depths, and output factors for each cone. Recommendations and correction factors published within the IAEA code of practice for the dosimetry of small fields (TRS 483) [2] were adopted for output factor determination. Beam data was collected using the Blue Phantom^2^ and OmniPro-Accept7 scanning software (IBA Dosimetry, Swarzenburg, Germany). Three different detectors were utilised; a stereotactic field diode (SFD) (IBA Dosimetry, Swarzenburg, Germany), microDiamond (PTW, Freiburg, Germany), and EBT3 GAFchromic™ film (Ashland, Bridgewater, USA). Specific data items and utilised equipment are listed in Table 1.

**Table 1:** Description of beam data acquired and detectors used for beam modelling

Beam data type	Measurement setup	Detector	Validating detectors
PDD	SSD: 90 cm; depth: 0–20 cm	IBA SFD	PTW MicroDiamond, EBT3 EBT3 GAFchromic™ Film
Profiles	SSD: 90 cm; depth: 1.5 cm, 5 cm, 10 cm, 20 cm	IBA SFD	EBT3 GAFchromic™ Film
Output factors	SSD: 90 cm; depth: 10 cm	IBA SFD/CC04	PTW MicroDiamond, EBT3 GAFchromic™ film

The jaws and MLC were set to a 6 × 6 cm square field for all cones.

### Water tank scanning

For PDD scans, the regions setup function in OmniPro-Accept7 was utilised to acquire step-by-step measurements for steps of 1 mm in the build-up region, and 2 mm beyond d_max_ to a depth of 20 cm. The detectors were carefully aligned to the beam’s central axis by measuring inline and crossline profiles at 1.5 and 20 cm depth. There was less than 0.4 mm lateral drift in detector position over this depth. The estimated uncertainty for reported d_max_ position (R100) and PDD values were 1 mm and 1% respectively for a k = 2 coverage factor.

Dose profiles were measured for all cone sizes at four depths (1.5 cm, 5 cm, 10 cm, and 20 cm). Of the scanning detectors available, the SFD had the smallest sensitive cross section in the plane perpendicular to the beam axis and was therefore used for all profile scans. The detector was aligned so that the stem was parallel to the beam axis as recommended [2]. A varying step-by-step resolution of 2 mm outside the field edge and 0.1 mm within penumbra was utilised. This was the limit in resolution for the Blue Phantom^2^ water tank. An uncertainty of 0.3 mm has been estimated for the reported full width of half-maximum (FWHM) and penumbra width data taken from these measurements, k = 2.

#### Output factors

All measurements were made at 10 cm depth, 90 cm SSD and normalised to a reference field size of 10 × 10 cm^2^. The intermediate field size chosen was that of the 39 mm cone as it fulfilled the requirement of maintaining lateral charged particle equilibrium across the ionisation chambers sensitive volume. The 6 MV beam had a TPR_20,10_ of 0.686 and the CC04 (IBA Dosimetry, Swarzenburg, Germany) chamber used at the intermediate and reference fields has a diameter of 4.8 mm. The relevant detector correction factors were taken from Table 26 published within TRS 483 [2] and linearly interpolated between data points as a function of equivalent square field size, $S_{clin}=\sqrt\pi\frac{FWHM}2.$

As with the PDD measurements, output factors were measured with two different solid state detectors and spot-checked with EBT3 film. The microDiamond and SFD detectors were positioned at 10 cm depth and centred within the beam profile. This was achieved by moving the detector in 0.1 mm increments until a peak in electrometer reading was found in both in-plane and cross-plane directions. Each repositioning of the detector was started from the same absolute coordinate to minimise positional uncertainty due to hysteresis. This method of ‘sweet spotting’ was repeated for all cones smaller than 13 mm in diameter, while for the larger cones, a common central coordinate was assumed. An uncertainty budget of 2% (k = 2) applies to these measurements.

#### EBT3 GAFchromic™ film measurements

**Fig. 1:**
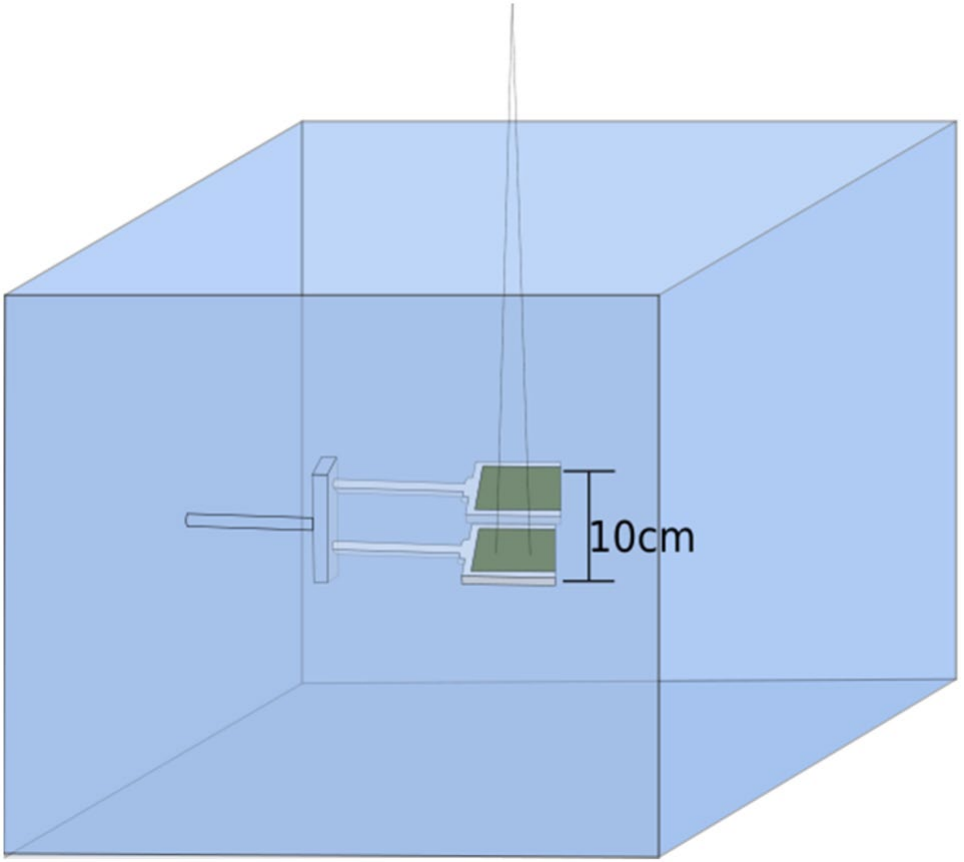
Schematic illustrating the setup of EBT3 film submerged in water used for PDD spot checks, output factor measurements and profiles

The PDDs, profiles and output factors were all spot checked for a subset of cone diameters (5 mm, 7 mm, 9 mm, 19 mm, and 39 mm) using EBT3 film (batch 03111902). To avoid any added uncertainty of water equivalence introduced by using solid water phantoms, 3D printed brackets were made so that two 5 × 5 cm film pieces, separated by 10 cm, could be accurately attached to the scanning system and submerged in water (Fig. 1). Considering that each film was only submerged for a few minutes and the analysed area was at least 5 mm away for the film edge in the worst case, it was deemed acceptable not to protect the film edges from water penetration. It has been shown that water will penetrate into the edges of EBT3 film by approximately 4 mm over a 24 h submersion time [10]. The films were calibrated at 1.5 cm depth and 100 cm SSD for a 10 × 10 cm^2^ field size during the same measurement session. Measurements were made at 1.5 cm, 10 cm, and 20 cm. All measurements were repeated 3 times and scanned at 75 dpi using an Epson 10000XL scanner in transmission mode 72 h post-irradiation. Analysis was made using the red channel and an ROI of 3 × 3 pixels (1.04 mm^2^) within a program developed on site. The uncertainty associated with all film measurements in this work has been estimated to be 4% (k = 2) [11].

### Beam modelling

RayStation TPS v9B offers two photon dose calculation engines, a Collapsed Cone Convolution (CCC) and Monte Carlo (MC) algorithm. Due to prior modelling experience and convenient calculation times, the CCC algorithm was used initially for beam modelling and served as a good starting point when utilising the MC dose engine. The modelling parameters and fluence computation above the patient plane are the same for both algorithms [12].

RayStation’s beam model has two different photon sources; the primary source—positioned at the Bremsstrahlung target with a small spot size and high intensity, and the scatter source—which has a relatively large spot size and low intensity, typically positioned close to the level of the flattening filter. The x and y dimensions for both are adjustable modelling parameters.

The photon spectrum is split into discrete bins where manual adjustment or inbuilt auto-modelling tools can be used to define the relative weights of each energy contribution. The auto modelling tools are only available for open field models and not cone collimated beams.

There is also a model for electron contamination originating from photon interactions in the linac head. This is represented by a parameterised equation in RayStation, $f\left(E\right)={E}^{C}{e}^{-E/{E}_{0}}$, where $C$ and ${E}_{0}$ are user-defined parameters.

Fluence variation away from the central axis is accounted for using radial scaling factors (beam profile correction), and spectral effects caused by the flattening filter are modelled by off-axis softening factors. Both of these can be adjusted as a function of radius away from the primary source.

Cone factor corrections are applied to scale the dose output at the reference depth as a function of cone size. The measured curves imported in to the TPS have an output defined by the absolute calibration multiplied by measured output factors. The purpose of cone factor corrections is to fine-tune the TPS calculated output so that the computed dose curves directly overlay the measured data at the reference depth.

### Model validation

#### Static beams, homogeneous phantom

Once modelling was complete, scanning data was verified within the planning module of the TPS as recommended by the AAPM medical physics practice guidelines (MPPG 5.a.) [13]. A virtual water phantom was used to simulate static beams at gantry 0° and 90 cm SSD so that PDDs, profiles, and output factors could be compared to measurement. A 1 mm dose grid and 0.1% ucertainty per beam were used for all calculations. The dose grid was aligned so that the beam’s isocentre was centred within a single voxel.

#### Arcs, homogeneous phantom

Treatment plans using stereotactic cones often invlove a 3D technique of non-coplanar partial arcs. Measurements of partial arcs about different phantom geometries were made and compared to TPS calculation. Arcs of 60 degrees (from gantry 330° to 30°) were delivered to the CC04 chamber positioned within the Blue Phantom^2^ at 10 cm depth introducing varying SSD and oblique incidence. 180° arcs (from gantry 270° to 90°) were delivered to a CC04 chamber housed within a StereoPHAN™ (Sun Nuclear, Melbourne, Florida). The chamber volume was simulated in the TPS by a 2 mm radius sphere and overridden to water to mitigate volume averaging and dose to medium discrepancies. The average dose within this structure was reported and compared to measurement. Only cones down to 13 mm diameter were measured with this method. EBT3 film was used for smaller fields and placed within Solid Water® (Gammex Inc., Middleton, US) slab geometry at three depths (3 cm, 5 and 10 cm), and the dose to a single voxel was reported. All calculations were made using a 1 mm^3^ dose grid and 0.3% uncertainty per beam. The uncertainty associated with measurements made by the CC04 has been estimated as a function of cone size. The cone size dependence of this budget includes the error associated with a 1 mm alignment discrepancy between the chamber and field centres, as well as the perturbation caused by the chamber within the radiation field. The dosimetric impact of alignment error was simulated via isocentre shifts in three dimensions within the TPS. The expected error introduced by not correcting for perturbation, has been estimated by dividing TRS 483 correction factors by calculated volume averaging factors. Volume averaging was estimated by simulating the CC04 in the TPS with a water sphere of 2 mm radius, and taking a ratio of the dose to this structure over that of a 1 mm^3^ voxel of water. The expanded uncertainty (k = 2) ranged from 2.5 to 4.8% for the 39 mm and 13 mm cone respectively.

### Inhomogenenous phantom

**Fig. 2:**
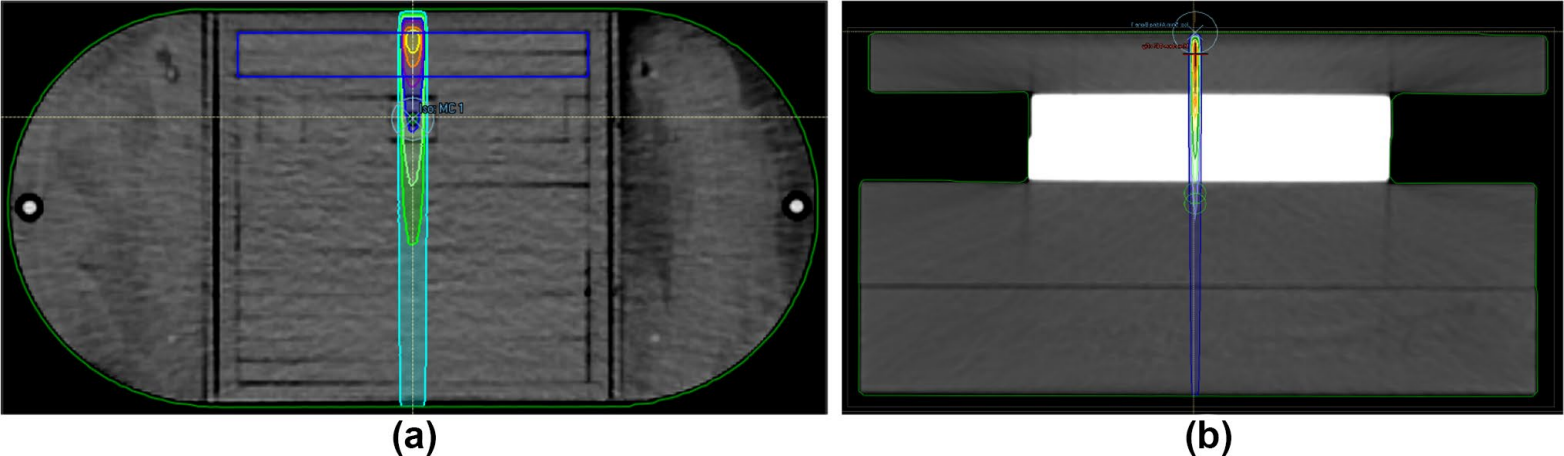
Different slab phantom geometries used to validate RayStation dose calculation. **a** I’mRT Phantom with blue contour’s material assigned to either air or bone for static beams and arcs using cones larger than 13 mm. **b** Slab phantom geometry containing Solid Water and bone used for the 5 mm cone. This phantom was needed so that EBT3 film could be placed within bone and at multiple depths immediately beyond the distal interface

Once calculations in water were verified, dose calculations in inhomogenous conditions were investigated. The I’mRT Phantom (IBA Dosimetry, Swarzenburg, Germany) allows different material inserts to be placed inside a water-equivalent universal body phantom. In this work, two simple geometries were used where a 2 cm thick inhomogeneity of bone analogue (Leeds Test Objects, Boroughbridge, UK) or air was introduced 1 cm below the phantom surface (Fig. 2a). The bone material had an average mass density of 1.93 g/cm^3^ which was assigned to the virtual material within the planning system. The virtual structure was created so that only a single CT dataset was necessary. The material assignments were based on bone (ICRP23) and air reference materials within the planning system so that appropriate elemental compositions and mean excitation energies would be used for the MC calculation (106.4 eV and 85.7 eV respectively). A CC04 chamber was placed 2 cm beyond the posterior interface (5 cm physical depth) and measurements were made for static beams at gantry 0° and arcs of 90° (from gantry 0° to 90°) for cones of diameter 13–39 mm.

To investigate the accuracy in the most extreme case of the 5 mm cone within bone and close to the interface, EBT3 film was utilised at multiple depths within slab geometry; mid-way through bone, at the distal interface, and three depths beyond (5 cm, 7 cm, 7.2 cm, 7.5 cm, and 8 cm physical depth respectively). The slab phantom depicted below had to be used instead of the IMRT phantom in order to establish the desired measurement depths (Fig. 2b). In this experiment there was no need for a virtual structure so the raw CT data was used for dose calculation and only a static beam from gantry 0° was investigated. It is worth noting that both detectors were calibrated within water equivalent materials and therefore measurements are reported as dose to water, while RayStation reports dose-to-medium with transport-in-medium in the case of the MC algorithm. One would therefore expect discrepancies between measurement and calculation of points within bone as investigated by Shaw et al. 2021 [14].

### End to end

As a final validation test, 5 treatment plans consisting of up to 10 non-coplanar conformal arcs were created and delivered to EBT3 film and an SRS MapCHECK® diode array housed inside a StereoPHAN™ phantom. The SRS MapCHECK® is a high density silicon detector array composed of 1013 SunPoint® 2 diode detectors with detector spacing of 2.47 mm, active detector area 0.48 × 0.48 mm^2^, detector volume of 0.007 mm^3^ covering an area of 77 × 77 mm^2^. The array is inserted in a 15 cm diameter head phantom known as the StereoPHAN™, which is composed of polymethyl methacrylate (PMMA). The phantom was aligned to the kV-imaging isocentre using cone-beam CT guidance and a 6-degree-of-freedom couch before true composite measurements were acquired in coronal and sagittal planes. Coincidence between the kV imaging and MV isocentres was verified via regular Winston-Lutz testing with a 1 mm tolerance as per AAPM MPPG 9a guidelines [15]. The measurements were compared to calculations made on a CT dataset of the phantom with 1 mm slices, 1 mm^3^ dose grid, and 0.3% statistical uncertainty. A water material of mass density 1.058 g/cm^3^ was assigned to the external contour as per department practice. The measurement vs. TPS calculation analysis was made using a global gamma comparison in absolute dose, with criteria of 1 mm/4% as per local protocol for SRS/SBRT plans, as well as with 1 mm/1% for comparison in the case of SRS MapCHECK measurements, all using a 10% dose threshold. The lower dose difference criteria was not used for EBT3 film measurements due to the relatively high uncertainty in absolute dose. The strict distance-to-agreement criteria is justified by the use of image guidance and the ability to align the film/calculated dose ditributions based on indentations made in the film by the StereoPHAN’s fiducial alignment markers. 1 mm is also the expected IGRT localisation accuracy for SRS [15]. Analysis was made within SNC Patient (Sun Nuclear, Melbourne, Florida) and VeriSoft (PTW, Freiburg, Germany) software for SRS MapCHECK and EBT3 film measurements respectively.

### Calculation time

Finally, an assessment of calculation time was made for a clinical, 10 arc plan as a function of both statistical uncertainty and cone diameter. A Python script was written and run within RayStation that reported dose computation time for each cone size with a fixed statistical uncertainty of 0.3% per plan. The script was then for calculations run on the same plan for two different cones (5 and 23 mm) while the statistical uncertainty was varied between 0.1 − 1.0%. A 22 × 18 × 24 cm^3^ dose grid size of 1 mm^3^ voxels containing a previous patient’s CT data was used for all calculations. All dose calculations in this work were run using a single Nvidia Quadro M6000, 24GB GPU (Nvidia, Santa Clara, California).

### Uncertainty budget

Uncertainty budgets have been generated following the guide to the expression of uncertainty in measurements (GUM) [16].

## Results

### Beam data

**Fig. 3:**
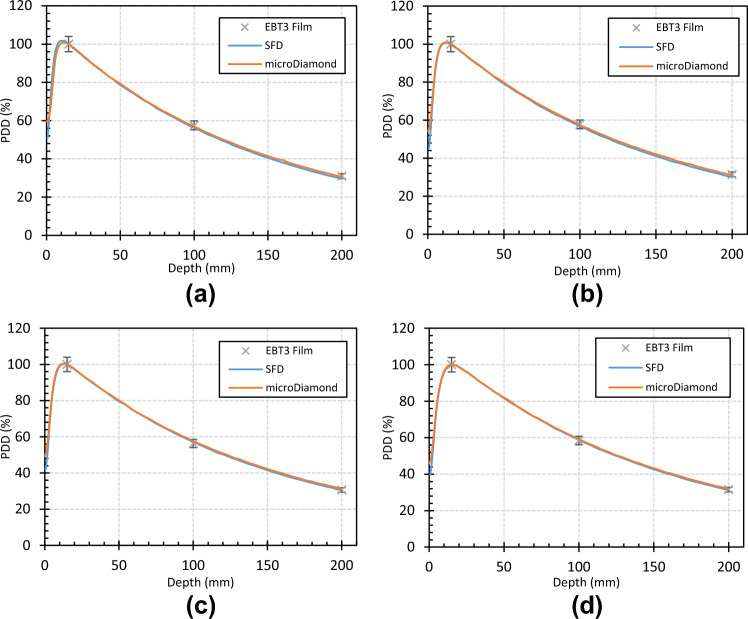
Percentage depth dose measured by the SFD and microDiamond detectors along with EBT3 film spot-checks for; **a** 5 mm, **b** 7 mm, **c** 9 mm, and **d** 19 mm diameter cones. Error bars of 4% (k = 2) are shown for the EBT3 film

Percentage depth dose data is presented in Fig. 3 and Table 2. microDiamond measurements were similar to that of the SFD with the largest discrepancies found for the smallest cones and at depth (Fig. 3). For the 5 mm cone, the microDiamond showed a higher response with a PDD value 1.4% greater than the SFD at 20 cm depth. This is within the standard uncertainty estimated for PDD measurements made with these detectors (1.0%, k = 2). The film spot checks were also in agreement with the solid-state detectors to within measurement uncertainty—the largest difference again found for the smallest cone. At 10 cm depth, the film gave a PDD value 2.5% higher than the SFD. The larger cones showed better agreement across all 3 detectors. Table 2 provides a summary of percentage depth dose results measured using the SFD.

**Table 2:** Depth of maximum dose and percentage depth dose at 5, 10, and 20 cm as measured by the SFD

Cone (mm)	R100 (cm)	d5 (%)	d10 (%)	d20 (%)
5	1.0	77.6	55.4	29.2
7	1.3	79.0	56.7	29.9
9	1.4	79.8	57.4	30.5
11	1.6	80.4	57.8	30.8
13	1.6	81.1	58.0	31.0
15	1.5	81.5	58.5	31.2
19	1.6	82.0	59.1	31.5
23	1.7	82.9	59.6	31.7
27	1.6	83.2	60.3	32.1
31	1.7	83.5	60.4	32.3
35	1.7	83.9	61.3	32.8
39	1.7	84.5	61.8	33.7

Uncertainties of 1 mm and 1% apply for R100 and PDD values respectively (k = 2)

**Fig. 4:**
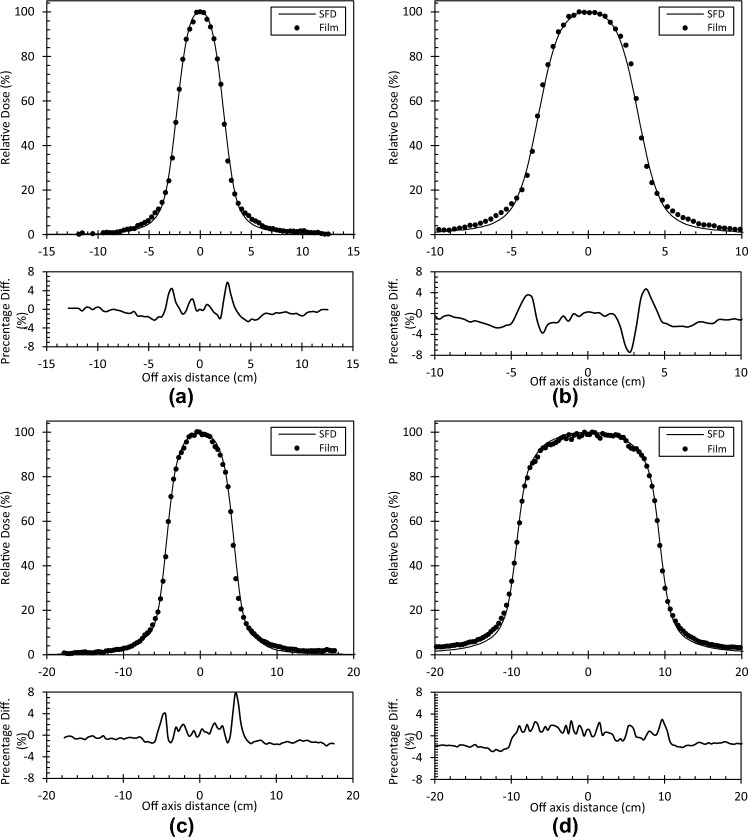
Crossline profiles measured at 10 cm depth by the SFD and EBT3 film and their point-by-point discrepancy. **a** 5 mm cone **b** 7 mm cone **c** 9 mm cone **d** 19 mm cone

Figure 4 illustrates crossline profiles measured at 10 cm depth by the SFD and EBT3 film for a 5, 7, 9, and 19 mm cone. It can be seen that the diode gave a similar penumbral shape to the film although less affected by noise. In all cases, it appears that the diode measured less out of field dose than the film and in the worst cases this was a 2% difference. In the penumbra, the local point-wise discrepancy was within 8%.

Excellent agreement to within 0.9% was found between the SFD and microDiamond output factor measurements across all cone sizes. An average was taken across both detectors for the final model with the film spot checks within 2.5% of these (Fig. 6; Table 3). This agreement is within the estimated uncertainty of 4.0% for film output factor measurements (k = 2).

**Table 3:** Output factors from SFD and microDiamond measurements for all cones along with EBT3 film spot-checks

Cone (mm)	SFD	microDiamond	EBT3 film	
5	0.488 ± 0.010	0.485 ± 0.010	0.499 ± 0.020	
7	0.582 ± 0.012	0.586 ± 0.012	0.583 ± 0.023	
9	0.649 ± 0.013	0.655 ± 0.013	0.649 ± 0.026	
11	0.695 ± 0.014	0.699 ± 0.014	–	
13	0.732 ± 0.015	0.734 ± 0.015	–	
15	0.759 ± 0.015	0.759 ± 0.015	–	
19	0.797 ± 0.016	0.797 ± 0.016	0.806 ± 0.032	
23	0.823 ± 0.016	0.823 ± 0.016	–	
27	0.842 ± 0.017	0.842 ± 0.017	–	
31	0.857 ± 0.017	0.856 ± 0.017	–	
35	0.868 ± 0.017	0.869 ± 0.017	–	
39	0.879 ± 0.018	0.879 ± 0.018	0.884 ± 0.035	

Measurements were at 10 cm depth, 90 cm SSD and relative to a 10 × 10 cm reference field

### Beam model

The secondary photon source, off-axis spectral adjustments, and MLC/jaw parameters had little to no effect on the model. Overestimation in surface dose was noticed for all cones so electron contamination was turned off via zero weighting to reduce this discrepancy. The final modelling process could be achieved in a few iterative steps;

- The Photon energy spectrum was adjusted manually until the computed PDD’s shape matched the measured curves over the full range of cone sizes.
- Primary source dimensions were altered until the gradient of beam penumbra matched measured curves.
- Adjustment of source dimensions and spectrum had a direct influence on dose output so the auto modelling cone factor correction algorithm was utilised to correct this. Manual tuning of cone factor corrections was needed when finalising the model.

**Fig. 5:**
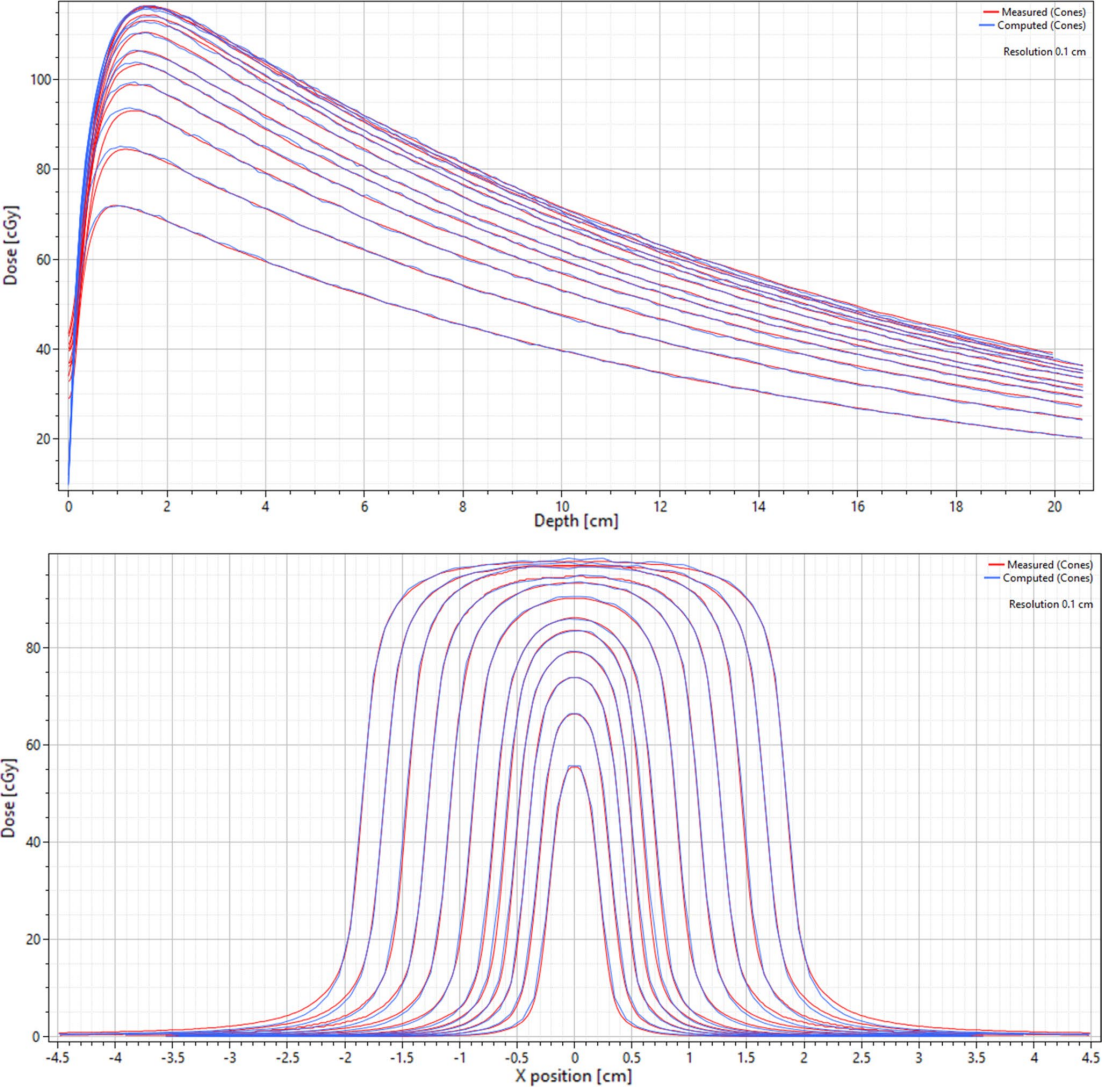
Screenshots taken from RayPhysics showing PDDs and profiles, measured vs. computed. The profiles are at 10 cm depth and calculations were made using the Monte Carlo dose engine

Following each machine model change, dose calculation has to be repeated. To increase calculation speed, the sample of cone sizes was reduced to a small fraction (4 over the full range). This also resulted in a far less busy display. Once satisfied with the fit, all remaining curves were imported and the final calculation was made using a 1 mm dose grid and statistical uncertainty of 0.3%. All of the important modelling parameters are presented in Table 4 and screenshots taken from RayPhysics are shown in Fig. 5 to illustrate the final model fit to measured data.

**Table 4:** Relevant modelling parameters from the final machine in RayPhysics

Energy spectrum		Primary source	Flattening filter and electrons	Cone factor corrections	
Energy (MeV)	Fluence (a.u.)	X and Y width (cm)	Weight	Diameter (cm)	Correction factor
0.5	0.05332	0.010	0.000	0.5	0.8950
1.0	0.17700			0.7	0.9180
1.5	0.07328			0.9	0.9360
2.0	0.04096			1.1	0.9470
2.5	0.03406			1.3	0.9565
3.0	0.03005			1.5	0.9580
3.5	0.03018			1.9	0.9660
4.0	0.02393			2.3	0.9750
5.0	0.02416			2.7	0.9750
6.0	0.01147			3.1	0.9850
7.0	0.00562			3.5	0.9830
				3.9	0.9850

### Model validation

#### Static beams, homogenous phantom

**Fig. 6:**
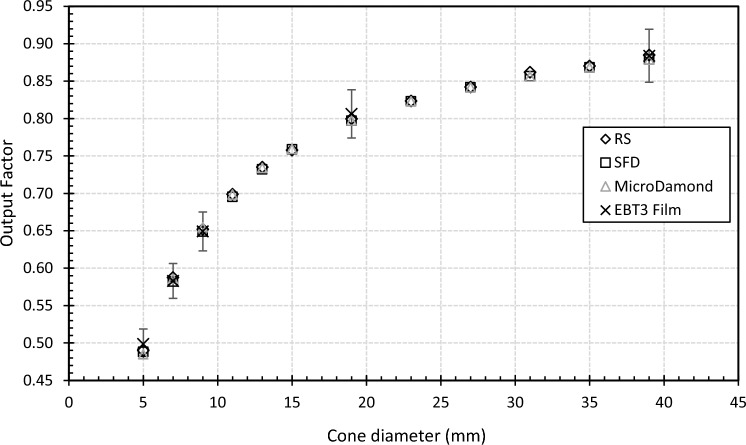
Output factors computed by RayStation (RS) compared to measurements using microDiamond, SFD, and EBT3 film

The PDDs could be matched very well across all cones beyond the point of maximum dose but discrepancies in the build-up region remained. From 1 mm depth to d_max_ the maximum single point discrepancy ranged from 10 to 21% across all cones while the average discrepancy was 4%. Beyond the point of maximum dose, the local point-by-point discrepancy was less than 1% for all cones. For profiles, the largest discrepancies found were out of field while the penumbra shape was very similar. Table 5 shows the calculated FWHM and penumbra widths for each cone with differences shown in mm. The field widths agreed with measurement to within 0.3 mm and penumbra widths to within 0.2 mm. For the field output factors, the TPS calculation agreed with measured data to within 0.7%, as seen in Fig. 6.

**Table 5:** FWHM and Penumbral width of crossline profiles at 10 cm depth

Cone size (mm)	TPS		SFD		TPS vs. SFD	
	FWHM (mm)	penumbral width (mm)	FWHM (mm)	penumbral width (mm)	ΔFWHM (mm)	Δ Penumbra (mm)
5	5.00	1.8	4.8	1.7	0.20	0.1
7	6.96	1.9	6.7	2.0	0.26	− 0.1
9	8.93	2.1	8.8	2.1	0.13	0.0
11	10.89	2.2	10.7	2.2	0.19	0.0
13	12.89	2.3	12.7	2.3	0.19	0.0
15	14.89	2.4	14.7	2.4	0.19	0.0
19	18.89	2.5	18.7	2.6	0.19	− 0.1
23	22.89	2.6	22.8	2.7	0.09	− 0.1
27	26.92	2.6	26.7	2.8	0.22	− 0.2
31	30.92	2.7	30.7	2.8	0.22	− 0.1
35	34.93	2.8	34.8	3.0	0.13	− 0.2
39	39.00	2.8	38.9	2.9	0.10	− 0.1

Comparison between TPS and SFD; An uncertainty of 0.3 mm applies to measured FWHM and penumbral width (k = 2)

### Arcs, homogenous phantoms

For 180° arcs about the stereoPHAN™, measurements using the CC04 chamber agreed to within 1.4% of calculation for cones larger than 13 mm (Table 6) which is inside the estimated uncertainty budget. Arcs of 60° about the water tank and solid water phantoms showed similar results with the largest discrepancy of 3.8% found for the 5 mm cone when comparing to EBT3 film at 5 cm depth (Table 7).

**Table 6:** Comparison between TPS calculation and IC measurements for 180° arcs around a stereoPHAN™

Cone diameter (mm)	Measured dose (cGy)	TPS (cGy)	Difference (%)
39	395.2 ± 9.9	390.2	− 1.3
35	390.4 ± 9.8	384.8	− 1.4
31	384.7 ± 9.6	383.1	− 0.4
27	377.9 ± 9.4	375.5	− 0.6
23	370.3 ± 9.3	365.4	− 1.3
19	356.8 ± 9.6	352.3	− 1.3
15	335.7 ± 10.7	334.7	− 0.3
13	320.4 ± 15.4	319.6	− 0.2

All beams delivered 500 MU at 100 cm SAD

**Table 7:** Comparison between calculation and IC measurements within the Blue Phantom^2^ for 60° arcs

Cone diameter (mm)	SSD (cm)	Depth (cm)	Monitor units	Measured dose (cGy)	TPS (cGy)	Diff. (%)
5^*^	97	3	200	115.3 ± 4.3	117.1	1.6
	95	5	200	99.4 ± 3.7	103.2	3.8
	90	10	500	202.9 ± 7.5	197.3	− 2.8
9^*^	97	3	200	149.8 ± 5.5	151.6	1.2
	95	5	200	132.7 ± 4.9	133.9	0.9
	90	10	500	267.5 ± 9.9	263.7	− 1.4
13^*^	97	3	200	163.6 ± 6.1	166.9	2.0
	95	5	200	150.9 ± 5.6	153.7	1.8
	90	10	500	292.7 ± 10.8	294.7	0.7
13	90	10	200	113.4 ± 5.4	116.7	2.9
15	90	10	200	118.4 ± 3.8	120.3	1.6
23	90	10	200	130.6 ± 3.3	131.2	0.5
31	90	10	200	136.5 ± 3.4	137.5	0.7
39	90	10	200	140.4 ± 3.5	141.6	0.9

^*^Measurements were made with EBT3 GAFchromic film within solid water. All other measured values were acquired with the ion chamber

### Inhomogeneity

Point doses in the I’mRT Phantom for static beams and arcs illustrated agreement with the RayStation planning system within the uncertainty estimated. The ion chamber measurements down to a 19 mm cone were all within 2.2% of calculation beyond bone and air inserts. For the 13 mm cone, this difference increased to 3.6% in the worst case which occurred beyond air (Table 8). The 90° arcs showed similar results as the TPS agreed with ion chamber measurements to within 2.9% (Table 9).

**Table 8:** Static beams at gantry 0° incident on the I’mRT Phantom containing air/bone inserts as depicted in Fig. 2

Cone diameter (mm)	Material insert	Measured dose (cGy)	TPS (cGy)	Diff. (%)
39	Bone	252.8 ± 6.3	252.8	0.0
27	Bone	243.1 ± 6.1	242.5	− 0.2
19	Bone	228.9 ± 6.2	230.1	0.5
13	Bone	207.0 ± 9.9	211.1	2.0
39	Air	284.3 ± 7.1	289.0	1.7
27	Air	276.6 ± 6.9	281.1	1.6
19	Air	263.6 ± 7.1	269.5	2.2
13	Air	240.8 ± 11.6	249.4	3.6

300 MU was delivered for all beams at 100 cm SAD

**Fig. 7:**
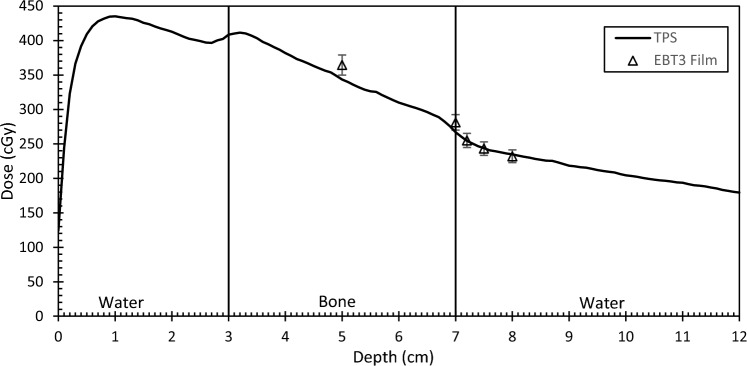
Central axis (CAX) dose as calculated by RS MC algorithm with point doses as measured by EBT3 film overlayed. Geometry as per Fig. 2

For the bone slab measurements using EBT3 film and the 5 mm cone, there were some obvious differences between measurement and calculation within bone and at the interface while agreement was observed immediately beyond the interface to within 1.2% (cf. Fig. 7). Inside bone, the TPS dose was 5.8% lower than the measurement. One should be reminded that there has been no attempt to account for the dose-to-medium discrepancies expected within bone nor has it been accounted for in the uncertainty budget. The error bars represent the 4.0% standard error (k = 2). Beyond the interface, agreement is within the error estimate.

**Table 9:** Arcs from 0–90° about the I’mRT Phantom containing air/bone inserts as depicted in Fig. 2

Cone diameter (mm)	Material insert	Measured dose (cGy)	TPS (cGy)	Diff. (%)
39	Bone	214.7 ± 5.4	213.2	− 0.7
27	Bone	204.9 ± 5.1	203.1	− 0.9
19	Bone	192.5 ± 5.2	192.2	− 0.2
13	Bone	173.2 ± 8.3	175.4	1.3
39	Air	248.4 ± 6.2	249.8	0.6
27	Air	240.5 ± 6.0	243.0	1.0
19	Air	228.3 ± 6.2	232.7	1.9
13	Air	207.2 ± 9.9	213.2	2.9

300 MU was delivered for all beams at 100 cm SAD

### End to end

Results of calculations of 10 clinical plans containing multiple non-coplanar arcs compared to true composite measurements in two planes are summarised in Table 10. No positional shifts were applied to the analysis as the stereoPHAN™ was precisely aligned with kV imaging isocentre as patients would be. One of the plans tested was that of a typical trigeminal neuralgia treatment using a 5 mm cone requiring EBT3 film. The global gamma pass rate was 98.9% and 98.7% in coronal and sagittal planes respectively for a 4%/1 mm criteria. The remaining plans utilising 19 mm, 15 mm, and 11 mm cones were measured using the SRS MapCHECK—results are summarised in Table 10.

**Table 10:** Global gamma pass rates with a 10% dose threshold measured with an SRS MapCHECK

Gamma criteria	Minimum pass rate (%)	Mean pass rate (%)
1%/1 mm	86.4	92.1
4%/1 mm	99.4	99.8

#### Calculation time

**Fig. 8:**
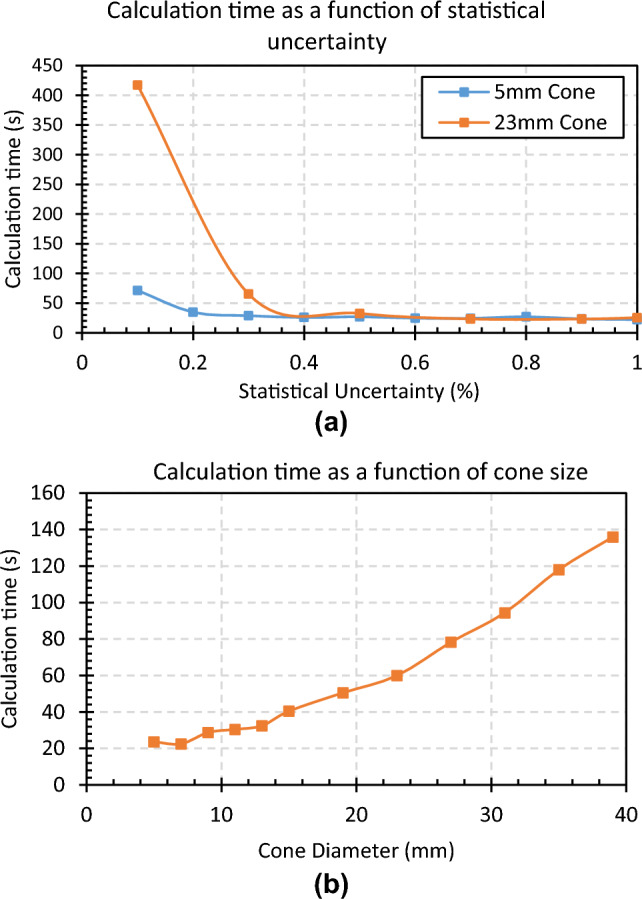
Top: Illustration of calculation time for two cone diameters with varying statistical uncertainty. Bottom: Calculation time with varying cone diameter and 0.3% uncertainty. The plan contained 10 non-coplanar arcs and was calculated on a 1 mm^3^ dose grid

For commonly used cone diameters ranging from 5 to 23 mm, calculation time was relatively constant down 1–0.3% statistical uncertainty as can be seen in Fig. 8. For statistical uncertainty below 0.3%, the calculation time increased substantially for larger cones. There is also an obvious correlation between cone size and calculation time as can be seen in Fig. 8 for a fixed uncertainty of 0.3%. For a plan containing 10 non-conformal arcs, a 1 mm dose grid, and 0.3% statistical uncertainty, the average calculation time was around 1 min across all field sizes. These results were found to be reproducible to within 3 s.

## Discussion

### Beam data

Beam data collection in the presence of cone collimated fields poses unique challenges and particular care during data collection is required. For PDD measurements the two major challenges are; (1) ensuring that the detector is aligned exactly with the beam’s central axis and (2) choosing an ideal detector. The former can be overcome via careful experimental practices. For the latter, one may refer to the literature where there is little prescriptive guidance because ultimately, the truly ideal detector doesn’t exist. The SFD has an active detector diameter of 0.6 mm compared to 2.2 mm for the microDiamond (when used parallel to the beam axis) which was considered an important advantage for the smallest cones to minimize concerns of changes in volume averaging as a function of depth. The microDiamond has the advantage of a very thin sensitive layer (1 μm) compared to 40 μm for the SFD, increasing its spatial resolution in the direction of a PDD scan. Solid-state detectors, including both unshielded diodes and synthetic diamond detectors, have been shown to demonstrate over-response in small fields. In the case of unshielded silicon diodes, it is understood that this is a result of the increased electron fluence and higher stopping power within the relatively dense sensitive layer [14–18], while for the microDiamond, it is suspected this is largely caused by the relatively dense layers encasing the sensitive volume [19], [20]. The concern with these attributes is that they are a function of energy and, therefore, potentially depth. This prompted the use of a third validating detector, EBT3 film, so that spot checks over the range of cone sizes could provide further confidence when finalising beam data.

For the PDD scans, the SFD and microDiamond were very similar, with the SFD response being slightly lower at depth for the smallest fields while the contrary occurred for larger cones—this potentially being a sign of overresponse to the higher proportion of low energy scatter present for the larger cones. For the smallest fields, the higher response of the microDiamond could possibly be due to diminishing levels of volume averaging with depth, remembering that this detector has an active diameter of 2.2 mm compared to 0.6 mm for the SFD. The differences, however, were small and well within the uncertainty of spot checks made by EBT3 film suggesting that either detector would have been appropriate. For profiles, the SFD gave results similar to EBT3 film except for the slight underestimation in out-of-field dose as mentioned previously. The local point-by-point discrepancies increased to as high as 8% in some instances but this was deemed acceptable in light of the steep dose gradients present in the penumbral region.

The output factors (Table 3) revealed excellent agreement between the two solid state detectors and illustrated the consistency that can be achieved when following TRS 483 methodology. The EBT3 film also showed agreement when considering the relatively large uncertainty associated with these measurements compared with the solid state detectors for the cones sampled. This ensured confidence in the final values used for modelling. There are currently no other publications using Aktina cones and flattened beams in the literature so the use of 3 detectors was justified here. One must take particular care with regards to hysteresis in the positioning system when sweet spotting as we found positioning the detectors from a common location had a substantial impact on readings. Finally, film still played an important role in data validation and the 3D printed brackets used to integrate the films with the water tank system proved to be useful and practically viable.

#### Beam model

Modelling within RayPhysics for a cone collimated beam was a relatively simple process with fewer parameters utilised compared to an MLC-based model. The greatest challenge was arriving at an optimised energy spectrum that gave a good fit across the cone sizes utilised in this work, especially around the build-up region. Mzenda et al. had similar issues in the build-up region for open field models and noticed discrepancies greater than 10% in some instances [7]. In this work, there is a systematic overestimation in dose within the build-up region as compared to measurement that could not seem to be improved within the model. This is most probably attributed to the relatively large low energy component used in the spectrum at 1 MeV (Table 4). The spectrum was optimised to give the best fit beyond d_max_ due to greater clinical significance. The contaminant electron and flattening filter sources were turned off completely via a weighting of 0 in an attempt to reduce the surface dose and the discrepancy from measurement in the build-up region. RayStation offers the option of having a maximum energy level within the spectrum that is different to the nominal beam energy. In our case, we added an energy bin at 7 MeV which seemed to add an extra degree of freedom, allowing a better match across all cone sizes. This is analogous to the work of Valdenaire et al. 2016 who extended their energy spectrum to 8 MeV and 12 MeV for 6FFF and 10FFF open field models respectively [21]. In the end, our final model was optimised for cones only and would not have been appropriate for larger open fields. It certainly seemed that it wouldn’t be possible to have a common model for cones and open fields in our case, with particularly different energy spectra and source size compared to other clinical beam models.

The speed of RayStation’s Monte Carlo dose engine made the process relatively painless but starting with a CCC model proved to be very beneficial in this work. Minimising the sample of cone sizes to 3 or 4 that covered the full range would also be recommended (5 mm, 9 mm, 21 and 39 mm cones were used in this work, for example). The auto modelling tools are limited for a cone model in RayStation. The output factor correction in auto modelling was useful but fine-tuning of these values was still necessary in the end.

#### Model validation

##### Homogeneous phantoms

Due to the nature of cone collimated treatments and the relatively simplistic dose distributions created, much of the validation is based on point dose measurements in varying geometry. The problem is that this is most practically acquired with an ionisation chamber which has a volume relatively large compared to the cones sampled. The sensitive volume of the CC04 chamber utilised in this work was represented within the planning system by a spherical ROI of a 2 mm radius. This simplistic representation is similar to that used by Scott et al. and should help negate issues of volume averaging but it still ignores all other perturbations caused by replacing the medium with a volume of air. Part of their study aimed to characterise the effect of detector density (not atomic number) on dose measured at central axis as a function of field size. It was shown that the response of an air filled ionisation chamber, of 1.45 mm radius, will decrease significantly for field widths roughly 1 cm or less even with volume averaging accounted for [22]. TRS 483 correction factors are available for the CC04 chamber for equivalent square field sizes 1.0 to 8.0 cm. These corrections are specific to output factor measurements made at 10 cm depth and other setup conditions outlined in the code of practice. These corrections were therefore not applied to the validation measurements of this work. The uncertainty added by not accounting for detector perturbation has been approximated by taking a ratio of the reported correction factors over the estimated volume averaging correction as a function of cone diameter. The assumption here is that by dividing out the volume averaging component from the correction factors we are left with the remaining detector perturbation correction. For cones smaller than 13 mm this was not possible as there are no correction factors reported within the code of practice for fields of this size and therefore uncertainty analysis was not able to be made with confidence. For these reasons, no measurements using the ionisation chamber have been reported for cones below this size. For homogeneous media and cones 13 to 39 mm in diameter, TPS calculations are in agreement to within 2% of the ion chamber measurements for a 95% confidence level (k = 2).

### Inhomogeneity

Inhomogeneities in the presence of small fields appear to be handled reasonably by the Monte Carlo algorithm. If we are to consider the results of the largest cone, where the ion chamber is most appropriately used, the agreement beyond bone and air is within 1%. The inclusion of gantry rotation did not introduce any discrepancies either. For the smallest cone of 5 mm, the use of EBT3 film was necessitated. In our department, this cone is used almost exclusively for the treatment of Trigeminal Neuralgia where a prescription of 75 Gy in a single fraction is given. The point where the nerve is targeted often immediately abuts bone for these patients so the accuracy of the algorithm in these conditions was of particular interest. As seen in Fig. 7, there is an increase in dose, as predicted by the algorithm, within bone at this field size. This could be explained by a re-establishment of charged particle equilibrium on CAX. In water, secondary electrons liberated from primary interactions at CAX can traverse beyond the field edge where they cannot be replaced. As the primary beam enters bone, secondary electrons will have a lower average path length in the higher density medium and therefore less likely to escape the field edge. Similar results have been reported by Parwaie and Mohammad et al. for their Monte Carlo studies of small fields and heterogeneous media [23, 24]. The film measurements resemble this effect, although inside bone the dose is substantially (5.8%) highr than predicted by the TPS. The aforementioned authors displayed the same results for EBT2 film measurements although quantification of the differences inside bone were not reported [23, 24]. One would suspect this to be partly attributed to dose to water vs. dose to medium differences reported by the film and algorithm respectively. Shaw et al. investigated this effect for spine SBRT cases [14]. They proposed conversion factors that could convert a film measurement (based on dose to water calibration) to measure dose consistent with planning systems that report dose to medium with transport in medium. The factor suggested for cortical bone (0.875) would decrease the film measurement by 12.5%, which in our cas, would give a discrepancy of 7.7% between measuremet and calculation in bone. However, Shaw’s study was generally based on MLC fields treating targets of around 4 × 4 cm, with the author suggesting that there may be some field size dependence on the proposed correction factors. With this being said, our findings showed that the planning system converges with the film measurements immediately beyond the distal interface. Here, there is agreement better than 1.2%—confirming the ccuracy of the algorithm in these scenarios. These results highlight the difficulties of verifying dose calculation accuracy in different materials using detectors calibrated in water for model-based algorithms. More research is needed in this area.

## End to end

The end-to-end tests using a stereoPHAN™ and SRS mapCHECK/EBT3 film detectors all passed with more than 95% (average of 99.6%) of points meeting a 1 mm/4% global gamma criteria. These measurements were made following phantom alignment using CBCT imaging and no further spatial adjustments were made within the software. Comparatively, Rose et al. conducted a multi-institutional validation study of the SRS mapCHECK array via patient specific QA (PSQA) measurements of 84 SBRT and SRS treatment plans across multiple planning systems and treatment units [25]. They found an average pass rate of 94.7% for global gamma comparisons with 3%/1 mm dose difference/distance-to-agreement criteria and a 10% lower dose threshold. The majority of treatment plans were based on VMAT plans of varying size and complexity. Brown et al. included end-to-end measurements using EBT-XD film for PSQA of a “virtual cone” technique using the HD-MLC on a Varian Edge linac (Varian Medical Systems, Palo Alto, CA). 7 treatment plans calculated using the Eclipse TPS’ Analytical Anisotropic Algorithm (AAA) v15.6 for Trigeminal Neuralgia plans showed a gamma passing rate of 100% for an absolute dose difference of 2% and distance-to-agreement of 1 mm with a 10% threshold [26].

### Calculation time

The speed of RayStation’s photon Monte Carlo dose calculation is satisfactory for cone based SRS treatment planning in the clinic. For cones of diameter less than 2 cm, the calculation time is relatively consistent down to a statistical uncertainty of 0.3% per plan. Below this, there is a sharp increase in calculation time. There is a direct correlation between field size and calculation time as one may expect so the choice of settings is somewhat dependent on the clinic’s needs. For us, an uncertainty of less than 0.5% was chosen with calculation time generally less than 2 min for a typical SRS treatment plan which is largely acceptable in the clinical context. For reference, Brown *et al’s.* study using the AAA algorithm, which is much less computationally demanding, showed an average calculation time of around 25 s for a Trigeminal Neuralgia plan using a 5 mm cone and around 30 min for a dynamic “virtual cone” technique [26]. In this work, a similar plan using the 5 mm cone could be calculated in under 30 s using the settings as mentioned above.

## Conclusion

It is stated within MPPG9a guidelines that key data points such as percentage depth dose and output factors for field sizes below 2 cm, should be compared to other machines with identical design [15]. This is advised as a means of identifying any gross errors that may arise due to the relatively intricate considerations necessary for small field dosimetry. The data presented in this work aims to serve this purpose and has been validated via the use of multiple commercially available detectors commonly used in the clinic. Beam modelling using RayStation’s Monte Carlo algorithm was achieved and the process has been summarised in a few simple steps. Small fields, as created by stereotactic cones, presented unique challenges that necessitated a stand-alone SRS beam model optimised for cones exclusively in this work. The clinical validation revealed satisfactory results as calculations compared to measurements within their estimated uncertainty for homogeneous and heterogeneous media using static beams and non-coplanar arcs. The GPU based Monte Carlo algorithm is relatively fast and facilitates a user friendly planning experience without compromising dose grid resolution or statistical uncertainty.

## References

[1] Schmitt D (2020). Technological quality requirements for stereotactic radiotherapy. Strahlenther Onkol.

[2] Palmans H, Andreo P, Huq MS, Seuntjens J, Christaki KE, Meghzifene A (2018). Dosimetry of small static fields used in external photon beam radiotherapy: summary of TRS-483, the IAEA–AAPM international code of practice for reference and relative dose determination. Med Phys.

[3] Wilke L (2019). ICRU report 91 on prescribing, recording, and reporting of stereotactic treatments with small photon beams: statement from the DEGRO/DGMP working group stereotactic radiotherapy and radiosurgery. Strahlenther Onkol.

[4] Ralston A, Liu P, Warrener K, McKenzie D, Suchowerska N (2012). Small field diode correction factors derived using an air core fibre optic scintillation dosimeter and EBT2 film. Phys Med Biol.

[5] Kawrakow I, Rogers DWO (2003) “The EGSnrc Code System: Monte Carlo Simulation of Electron and Photon Transport”. System. pp. 2001–2003,

[6] Richmond N, Angerud A, Tamm F, Allen V (2021). Comparison of the RayStation photon Monte Carlo dose calculation algorithm against measured data under homogeneous and heterogeneous irradiation geometries. Physica Med.

[7] Mzenda B, Mugabe KV, Sims R, Godwin G, Loria D (2014). Modeling and dosimetric performance evaluation of the RayStation treatment planning system. J Appl Clin Med Phys.

[8] Chen J (2019). Optimizing beam models for dosimetric accuracy over a wide range of treatments. Physica Med.

[9] Lee YC, Kim Y (2021). Commissioning of the TrueBeam STx 6 MV FFF Beam in the RayStation Treatment Planning System for SRS and SBRT treatments. Int J Med Phys Clin Eng Radiat Oncol.

[10] León-Marroquín EY, Lárraga-Gutiérrez JM, Herrera-González JA, Camacho-López MA, Villarreal JE, Barajas, García-Garduño OA (2018). Investigation of EBT3 radiochromic film’s response to humidity. J Appl Clin Med Phys.

[11] Aldelaijan S, Devic S (2018). Comparison of dose response functions for EBT3 model GafChromic™ film dosimetry system. Physica Med.

[12] RaySearch Laboratory (2019). RAYSTATION 10A Reference Manual.

[13] Geurts MW (2022). AAPM MEDICAL PHYSICS PRACTICE GUIDELINE 5.B: commissioning and QA of treatment planning dose calculations—megavoltage photon and electron beams. J Appl Clin Med Phys.

[14] Shaw M (2021). Measuring the dose in bone for spine stereotactic body radiotherapy. Physica Med.

[15] Halvorsen PH (2017). AAPM-RSS Medical Physics Practice Guideline 9.a. for SRS-SBRT. J Appl Clin Med Phys.

[16] ISO GUM (2008). “ISO/IEC GUIDE (2008) 98-3: 2008, Guide to the expression of uncertainty in measurement”.

[17] Qin Y, Zhong H, Wen N, Snyder K, Huang Y, Chetty IJ (2016). Deriving detector-specific correction factors for rectangular small fields using a scintillator detector. J Appl Clin Med Phys.

[18] Cranmer-Sargison G, Weston S, Evans JA, Sidhu NP, Thwaites DI (2011). Implementing a newly proposed Monte Carlo based small field dosimetry formalism for a comprehensive set of diode detectors. Med Phys.

[19] Ralston A, Tyler M, Liu P, McKenzie D, Suchowerska N (2014). Over-response of synthetic microDiamond detectors in small radiation fields. Phys Med Biol.

[20] Looe HK, Delfs B, Poppinga D, Jiang P, Harder D, Poppe B (2018). The ‘cutting away’ of potential secondary electron tracks explains the effects of beam size and detector wall density in small-field photon dosimetry. Phys Med Biol.

[21] Valdenaire S, Mailleux H, Fau P (2016). Modeling of flattening filter free photon beams with analytical and Monte Carlo TPS. Biomed Phys Eng Express.

[22] Scott AJD, Kumar S, Nahum AE, Fenwick JD (2012). Characterizing the influence of detector density on dosimeter response in non-equilibrium small photon fields. Phys Med Biol.

[23] Parwaie W, Geraily G, Mehri-Kakavand G, Babaloui S, Rezvani S, Pursamimi M (2022). Dosimetry of small photon fields in the presence of bone heterogeneity using MAGIC polymer gel, gafchromic film, and Monte Carlo simulation. Rep Practical Oncol Radiother.

[24] Mohammad A, Nedaie HA, YarAhmadi M, Banaee N, Naderi M, Tizmaghz Z (2014) Dosimetric evaluation of heterogeneities in small circular fields of 6 MV photon beams with EBT2 and EDR2 Films: comparison with Monte Carlo Calculation. J Modern Phys 05(16):1608–1616. 10.4236/jmp.2014.516162

[25] Rose MS (2020). Multi-institution validation of a new high spatial resolution diode array for SRS and SBRT plan pretreatment quality assurance. Med Phys.

[26] Brown TAD, Ayers RG, Popple RA (2022). Commissioning a multileaf collimator virtual cone for the stereotactic radiosurgery of trigeminal neuralgia. J Appl Clin Med Phys.

